# Decrease of peripheral blood mucosal‐associated invariant T cells and impaired serum Granzyme-B production in patients with gastric cancer

**DOI:** 10.1186/s13578-020-00518-9

**Published:** 2021-01-09

**Authors:** Chunyan Shao, Chenwen Zhu, Yun Zhu, Jiqing Hao, Yongxiang Li, Huaqing Hu, Li Si, Fei Zhong, Xuefu Wang, Hua Wang

**Affiliations:** 1 Department of Oncology, The First Affiliated Hospital of Anhui Medical University, Hefei, 230022 China; 2 Department of Oncology, Fuyang Hospital of Anhui Medical University, Fuyang, 236000 China; 3 Department of Gastrointestinal Surgery, The First Affiliated Hospital of Anhui Medical University, Hefei, 230022 China; 4 Health Management Center, The First Affiliated Hospital of Anhui Medical University, Hefei, 230022 China; 5 Department of Clinical Laboratory, The First Affiliated Hospital of Anhui Medical University, Hefei, 230022 China; 6 School of Pharmacy, Inflammation and Immune Mediated Diseases Laboratory of Anhui Province, Hefei, 230032 China; 7 Institute of Liver Diseases, Anhui Medical University, Hefei, 230032 China

**Keywords:** MAIT cells, Gastric cancer, Immunotherapy, Immune surveillance, IFN-γ, TNF-α

## Abstract

Mucosal-associated invariant T (MAIT) cells are an invariant T cell subset, which have been reported to play an antimicrobial role in infectious diseases. However, little is known about it in malignant diseases and tumors, especially in gastric cancer (GC). So in this study, we aim to examine the frequency, phenotype, partial functional capacity and clinical relevance of this cells from GC patients’ peripheral blood by flow cytometry. It was shown that the frequency of peripheral blood MAIT cells was negatively correlated with their increasing age in healthy adults. Importantly, comparing to the healthy controls (HC), the frequency and the absolute number of MAIT cells from GC patients’ peripheral blood with or without chemotherapy were both significantly lower than those. For the phenotype, the proportion of CD4^−^MAIT cell subset in GC patients without chemotherapy was lower than in HC, but higher than in GC patients with chemotherapy. Whereas, the proportion of CD4^−^CD8^+^MAIT cell subset in GC patients without chemotherapy was significantly lower than that in HC. Finally, the level of Granzyme-B (GrB), a molecule associated with MAIT cells was markedly lower in GC patients. But the correlation between the serum levels of GC-associated tumor antigens and the percentages of MAIT cells in GC patients was not observed. In conclusion, our study shows the decreased frequency, changed phenotypes and partial potentially impaired function of MAIT cells in GC patients, suggesting a possible MAIT cell-based immunological surveillance of GC.

## Introduction

Mucosal associated invariant T (MAIT) cells, a population of lymphocytes, are a kind of key immune cells that was previously discovered but has recently once again become a focus of medical research. It comprises up to 1–10% of peripheral blood total T cells in humans [1]. This cells are innate-like T cells that expressing a semi-invariant T cell receptor (TCR) of Vα7.2^−^Jα33 chain and a limited array of Vβ2 or Vβ13 chain in humans [2]. In addition to the Vα7.2 TCR, MAIT cells are also characterized by a high expression of NK cell receptor CD161 and IL-18Rα on cell surface [2–4]. In contrast to conventional T cells, MAIT cells recognize antigens bounded with major histocompatibility complex (MHC) molecules, they also recognize bacterial derived riboflavin (vitamin B2) metabolites presented by the MHC class 1b-like related protein (MR1) [5]. They are abundant in humans and especially enriched in mucosal layers, such as gastric mucosa, the usual site of tumor occurrence and development. This cells have been found both within primary and metastatic tumors [6]. But now, whether they can hasten malignancy or regulate to anti-cancer immunity is unclear.

In human clinical diseases, gastric cancer (GC) is one of leading causes of cancer‑related mortality worldwide and is a notable disease due to its heterogeneity [7]. GC usually destroys the mucosal homeostasis and barrier defense function. The development and prognosis of GC are also closely associated with the mucosal immunity in the body. So mucosal immune cells play an important role in gastric cancer. Although the incidence and mortality rate of GC have decreased overall in recent years, it also remains the major cause of cancer‑related mortality in developing countries [8] and seriously affects the healthy and life safety of humans. Tumor immunotherapy is a new method for tumor treatment after the traditional surgery, chemotherapy and radiotherapy. Immunotherapy is typically more tumor-specific and less toxic than chemotherapy, has brought renewed hope to many cancer patients, including those with GC. Once successfully implemented, immunotherapeutic modalities may narrow down the tumor mass, preparing for other treatments or even preventing postoperative relapse. With the further understanding of tumor microenvironment and tumor escape mechanism, it has become a new and of academic value research direction to mobilize the immune system to resist tumor.

Previous studies have shown the relationships between different types of T cells and the progression of GC [9, 10]. It has been recognized for a long period that unconventional T cells also can promote tumor rejection, and there are reasons why it is of value to translate these researches into clinical trials. Unconventional T cells offer several advantages that may help to improved T cell immunotherapy for human cancer [11]. MAIT cells have already been suggested to play a key role in immune surveillance of tumor via the contact response [12]. And MAIT cells have been reported in many studies in colorectal cancer (CRC), but rarely in GC [12–15]. We hypothesize that MAIT cells may also provide an attractive therapeutic target in GC. So, our work have assessed the frequencies, the partial functional capacities, and the clinical relevance of MAIT cells in peripheral blood of GC patients. Our findings indicated that MAIT cells did show significant differences between GC patients and normal controls, and could provide valuable experimental basis for immunotherapy researches of cancer.

## Materials and methods

### Human samples

The samples were peripheral blood, which came from 164 healthy adults (as healthy controls) and 87 GC patients enrolled at the First Hospital of Anhui Medical University, Hefei, China from November 2018 to May 2019. The healthy controls included in this study were all from the physical examination center, without tumor history, autoimmune disease, or recent acute infection history. The patients with GC were diagnosed by histological examination of biopsied tumor tissues obtained from gastroscopy.

### Isolation of PBMCs and stimulation of MAIT cells

Peripheral blood mononuclear cells (PBMCs) were isolated from the peripheral blood by gradient centrifugation with Ficoll-Paque Plus reagents (P8610, Solarbio, China), following the manufacturer’s protocol. To assess intracellular cytokine production, the CD3^+^TCRγδ^−^Vα7.2^+^CD161^+^ MAIT cells were stimulated with phorbol 12-myristate 13-acetate (PMA), ionomycin and monensin for 4–6 h in RPMI-1640 medium (SH30809.1, America) supplemented with 10% heat-inactivated FBS, placed in the cell incubator with 37 °C and 5% CO_2_.

### Flow cytometry

Single-cell suspensions were stained with the fluorescent-labeled antibodies (anti-human series, Biolegend), including FITC-CD3(OKT3), PE-TCR/γδ(B1), APC- TCRVα7.2(3C10), Brilliant Violet 510-CD161(HP-3G10), PE/Cy7-IFN-γ(B27), PE/Cy7-IL-2(MQ1-17H12), Brilliant Violet 421-TNF-α(MAb11), Brilliant Violet 421-IL-17A(BL168), PE/Cy7-CD4(RPA-T4), APC/Cyanine7-CD8(SK1). Isotype controls were used to determine cut-off levels for positive staining. Data were acquired using Flow Cytometer (Backman, America) and analyzed by FlowJo_V10 software.

### Detection of serum cytokines and GrB by CBA kit

The samples were human serum. Five kinds of Capture Bead mixtures were prepared according to the Instruction Manual of Cytometric Bead Array (CBA) Kit (BD, America). Each kit must make a standard curve. Standard tubes and negative control were performed for each experiment. In the test tube, added 5 kinds of Capture Bead mixtures (IL-2, IL-17A, IFN-γ, TNF-α and GrB) and PE (phycoerythrin) fluorescence Detection Reagent 50 µl for each, and then added 50 µl serum into the test tube for 3 h in the dark, RT. Before detection on the machine, added 1 ml Wash Buffer of to each tube, centrifuging for 200×*g*, 5 min. Flow Cytometer (Backman, America) was used for detection. FCAP Array v3 was used for data analysis.

### Statistical analysis

Data are expressed as mean values unless specified. The differences between groups were analyzed by paired or unpaired *t* tests. Other data were analyzed by the two-way ANOVA followed by post hoc Bonferroni tests using the Prism Version 5 (GraphPad) and SPSS Statistics 20. The potential correlation between variables was analyzed by the Spearman rank correlation test. Data is expressed as x ± s. The *p* values < 0.05 were considered to be statistically significant.

## Results

### Frequency of MAIT cells from healthy adults peripheral blood

**Fig. 1:**
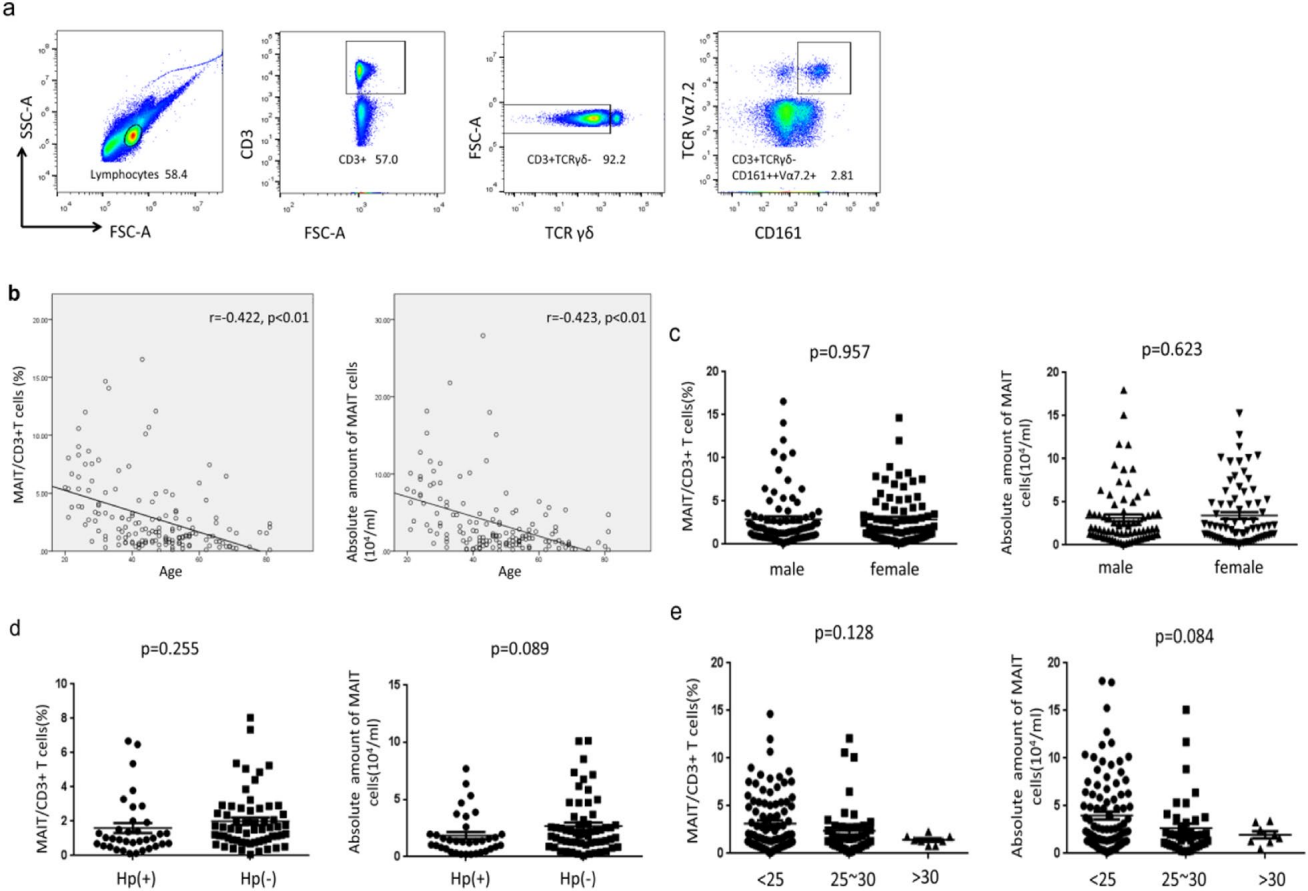
MAIT cells distribution in healthy adults. Samples were obtained from 164 healthy adults and stained with fluorescent antibodies against CD3, CD161, TCRγδ and TCRVα7.2. MAIT cells frequency was characterized by flow cytometry. These cells were gated on living lymphocytes for first, then on CD3^+^TCRγδ^−^ T cells, and finally on CD3^+^TCRγδ^−^Vα7.2^+^CD161^+^ T cells. The percentages of CD3^+^TCRγδ^−^Vα7.2^+^CD161^+^ MAIT cells in CD3^+^ T cells were determined. **a** Flow cytometry analysis of MAIT cell. In peripheral blood from healthy adults, age (**b**) and gender (**c**) were both related to the percentage of MAIT/CD3^+^ T cells and the absolute number of MAIT cells (104/ml), while, Hp infection (**d**) and BMI (**e**) were neither dependent of the percentage of MAIT/CD3^+^ T cells and the absolute number of MAIT cells (104/ml)

In previous studies, it has been shown that CD3^+^TCRγδ^−^Vα7.2^+^CD161^+^ T cells can be considered as MAIT cells [1]. Accordingly, in this article, we defined and counted the MAIT cells as CD3^+^TCRγδ^−^Vα7.2^+^CD161^+^ T cells. PBMCs were isolated from 164 healthy adults (male n = 83, female n = 81, the ages range from 20 to 81 years) and stained with fluorescent antibodies against CD3, CD161, TCRγδ and TCR Vα7.2. As shown in Fig. 1a, the frequency of CD3^+^TCRγδ^−^Vα7.2^+^CD161^+^ T cells among total CD3^+^ T cells in individuals were determined by flow cytometry. We concluded the percentage of MAIT cells in peripheral blood were negatively correlated with increasing age (*r* = − 0.422, *p* < 0.01, n = 159) in healthy adults. A similar trend was observed in the absolute number of MAIT cells (10^4^/ml) (*r* = − 0.423, *p* < 0.01, n = 151) (Fig. 1b). But no significant differences were detected between men and woman in the percentage (male 2.88%/female 2.86%, *p* = 0.957, n = 164) and absolute amount (male 3.16/female 3.44, *p* = 0.623, n = 149) of MAIT cells (10^4^/ml) in peripheral blood from healthy adults (Fig. 1c). In past reports, Lee et al. manifested the circulating MAIT cell levels were found to vary widely (0.19–21.7%) in humans and the levels in elderly subjects (age, 61–92 years) were significantly lower than in young (age, 21–40 years). They also pointed out that no difference was detected in the circulating MAIT cell levels between male and female subjects [16]. Their conclusion further supports our findings.

Expect for the separate analysis of age and gender, we also collected the information of *Helicobacter pylori* (Hp) infection and body mass index (BMI) of healthy donors and compared them in groups. As shown in Fig. 1d, e, we found that these factors had no effect on the proportion and absolute number of MAIT cells in the peripheral blood of healthy people.

### Difference in MAIT cells’ frequency between GC patients and healthy subjects

As shown in previous reports, MAIT cells’ frequency were changed with age [1, 16], our data also proved this view (Fig. 1b). Therefore, it is necessary to match the age and gender in each study. According to epidemiological researches, people who aged > 45 years to have GC was twice as likely as younger [17]. And the incidence of GC in male was approximately twice as high as female [18]. So, we firstly matched the age (mostly > 45 years old) and gender (male:female = 2:1) on the basis of the epidemiological trends in GC, as Table 1.

**Table 1:** Clinical characteristics of GC patients

Characteristics	GC without chemotherapy (n = 38 )	GC with chemotherapy (n = 49 )	P value
Age (years)			
≤ 45	3	3	
> 45	35	46	
${\bar{\text{x}}} \pm {\text{s}}$	61.42 ± 1.85	60.98 ± 1.47	0.85
Gender			
Male	26	34	
Female	12	15	
M:F	26:12	34:15	0.923

**Fig. 2:**
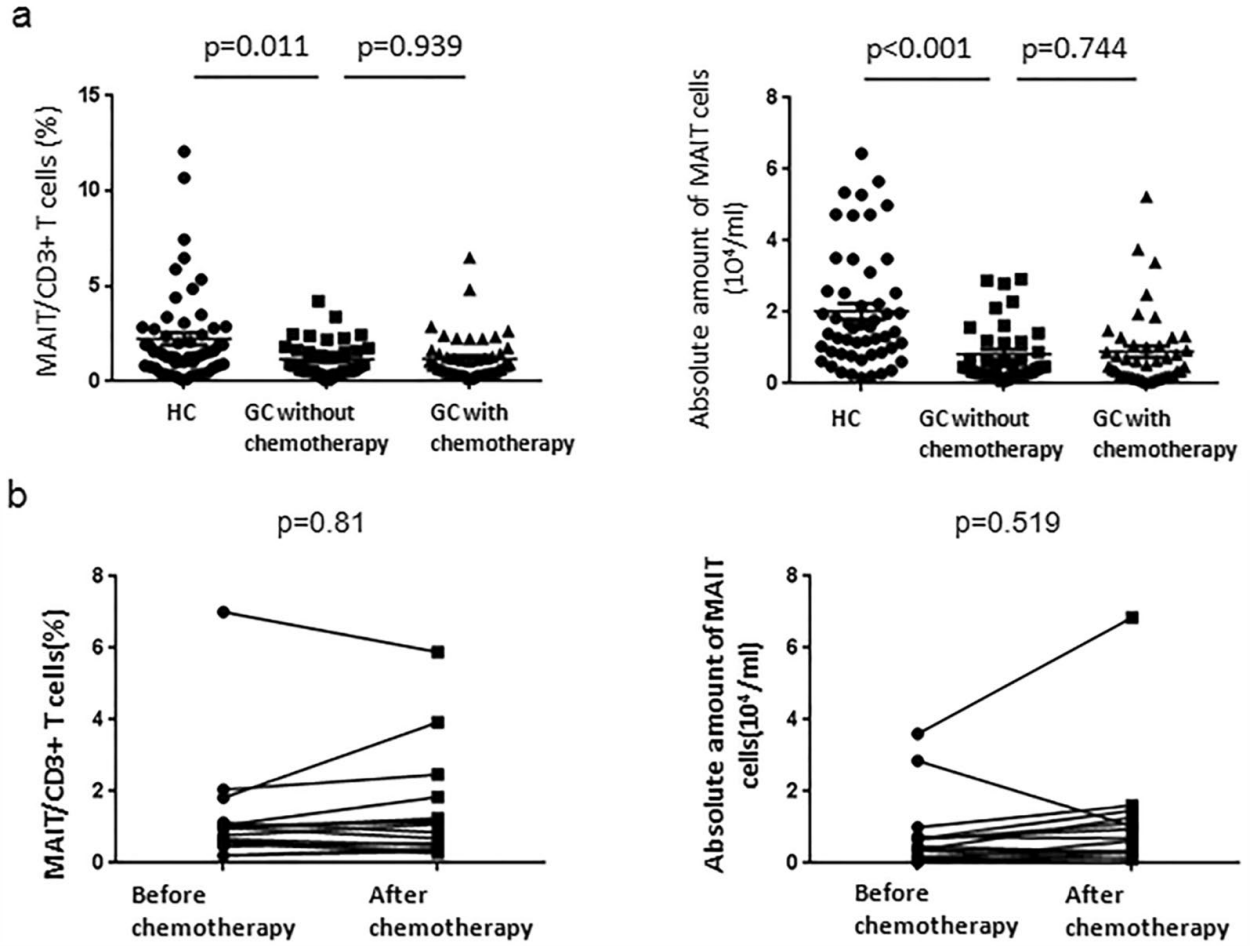
Proportion and absolute number of MAIT cells in GC and HC. Total of 87 GC patients (49 cases with chemotherapy, 38 cases without chemotherapy) and 56 HC, which gender- and age-matched, provided the research samples. In addition, 16 samples were obtained from GC patients to determine the percentage of MAIT cells among CD3^+^ total T cells before and after chemotherapy. The CD3^+^TCRγδ^−^Vα7.2^+^CD161^+^ MAIT cells were determined by fluorescent assays. **a** Differences in the proportion and absolute number of MAIT cells (10^4^/ml) in peripheral blood from GC patients and healthy controls. Moreover, the proportion and absolute number of MAIT cells (10^4^/ml) from GC patients’ peripheral blood were compared with or without chemotherapy. **b** Frequency and absolute number of MAIT cells in the same GC patients before and after chemotherapy

From results, we found that the percentage of MAIT cells in peripheral blood from GC patients were significantly lower (mean 1.17%) than that in HC (2.24%, *p* = 0.011). The absolute number of MAIT cells (10^4^/ml) was also the same trend (2.02 in HC and 0.82 in GC without chemotherapy, *p* < 0.001). In contrast, there were no significant differences in percentage and absolute amount of MAIT cells (10^4^/ml) from GC patients with or without chemotherapy (Fig. 2a). There was also no significant changes in the percentage and absolute number of MAIT cells in GC patients before and after chemotherapy (Fig. 2b). So we guess the frequency of MAIT from GC patients peripheral blood is unaffected by chemotherapy.

### Flow cytometry analysis of MAIT cells’ different subsets in GC patients

**Fig. 3:**
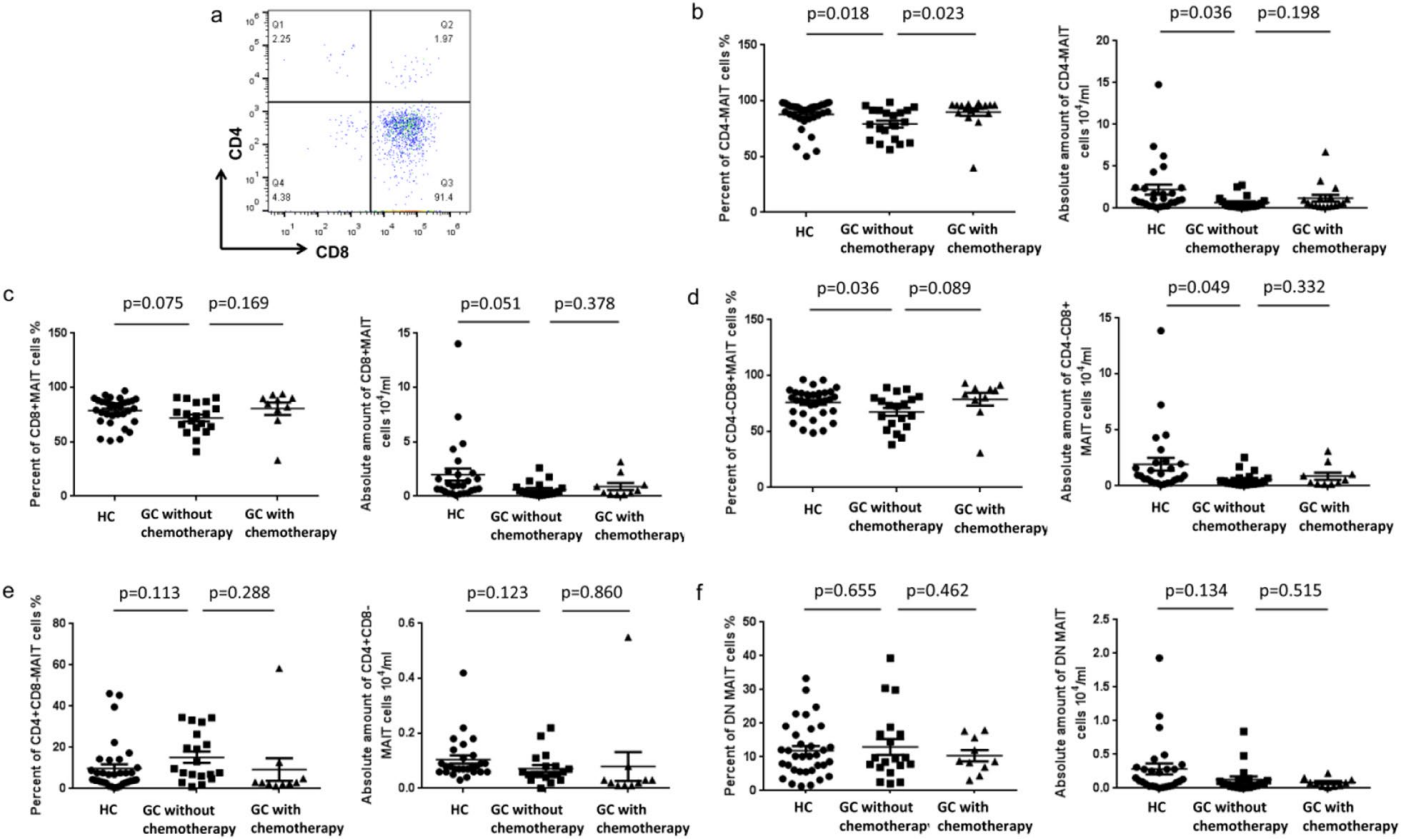
Differences in CD4 and CD8 phenotypes of MAIT cells in GC patients and HC. The different subsets of circulating MAIT cells were gated on CD3^+^TCRγδ^−^Vα7.2^+^CD161^+^ MAIT cells and the percentages of CD4^−^CD8^+^, CD4^+^CD8^−^ or CD4^−^CD8^−^(DN) MAIT cells were determined. **a** The gating strategy and the flow cytometry analysis of the different subsets of MAIT cells. **b**–**f** Differences with CD4 and CD8 phenotypes in MAIT cells between patients with GC patients and HC, and among the GC patients between with or without chemotherapy

Based on the expression of CD4 and CD8 phenotypes, MAIT cells populations can be divided into three main subsets: CD4^−^CD8^+^, CD4^+^CD8^−^ and CD4^−^CD8^− ^(double-negative, DN) subsets [16, 19]. Analysis of circulating MAIT cells in GC patients and HC indicated that most of MAIT cells were either CD8^+^ or DN phenotype, and there was a very little proportion of CD4^+^ subset (Fig. 3a).

The percentage of CD4^−^ MAIT cells in total MAIT cells from the GC patients without chemotherapy (79.2%, *p* = 0.018) were significantly lower than from HC (87.8%, Fig. 3b left). And in the gastric cancer groups, the proportion of CD4^−^ MAIT cells without chemotherapy (79.2%, *p* = 0.023) was lower than that with chemotherapy (89.8%, Fig. 3b left), the absolute amount of CD4^−^ MAIT cells with chemotherapy (2.23, *p* = 0.036) was also lower than that without chemotherapy (0.67, Fig. 3b right).

In addition, the GC patients (67.4%, *p* = 0.036) was significantly lower than the HC (75.8%, Fig. 3d left) in CD4^−^CD8^+^ MAIT cells. The corresponding absolute amount of CD4^−^CD8^+^ MAIT cells show the same differential trend (mean 1.94/0.57, p = 0.049, Fig. 3d right). But the percentages of MAIT cells in other groups showed no differences.

### Partial functional capacity of MAIT cells in GC patients

**Fig. 4:**
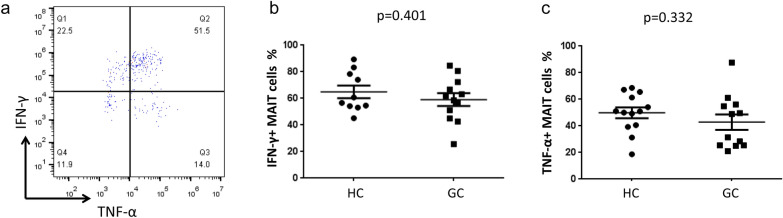
Assays of partial MAIT cells’ functional capacities. **a** MAIT cells were stimulated with PMA + ionomycin in vitro and detected by flow cytometry. Proportion of IFN-γ^+^ MAIT cells (**b**) and TNF-α^+^ MAIT cells (**c**) in peripheral blood from GC patients and HC

The present studies reported that MAIT cells exhibited wide-ranging functional impairments, which depended on their physical location but not chemotherapy [13]. To knowledge the partial cytokine-secretion function of peripheral blood MAIT cells, the isolated cells were stimulated with PMA (50 ng/ml) and ionomycin (1 μg/ml) in-vitro in complete medium for 4–6 h, then, detected by flow cytometry. From the data, both IFN-γ^+^ MAIT cells (*p* = 0.401) and TNF-α^+^ MAIT cells (*p* = 0.332) were all having no significantly differences between GC patients and HC (Fig. 4b, c).

### Correlations between circulating MAIT cells and clinical relevance

The levels of serum CEA, CA19-9 and CA72-4 remain a valuable biomarker for the evaluation of GC progression [20, 21]. To determine whether the serum levels of these tumor markers are associated with MAIT cells’ frequency, we collected relevant information and made a comparative analysis. From the comparison, we knew the frequency of MAIT cells had no significant correlations with serum CEA, CA19-9 and CA72-4 levels, in Table 2.

**Table 2:** Clinical tumor marks information of GC patients

Tumor marks	Number (n )	MAIT cells frequency, mean values (${\bar{\text{x}}} \pm {\text{s}}$)	P value
CEA (µg/l)			
≤ 5	20	1.10 ± 0.24	0.451
> 5	17	1.43 ± 0.39	
CA19-9 (U/ml)			
≤ 37	27	0.87 ± 0.13	0.078
> 37	8	1.42 ± 0.34	
CA72-4 (ng/ml)			
≤ 3.3	13	1.10 ± 0.35	0.594
> 3.3	22	1.36 ± 0.30	

### The levels of MAIT cell‐associated cytokines and GrB in GC patients serum

**Fig. 5:**
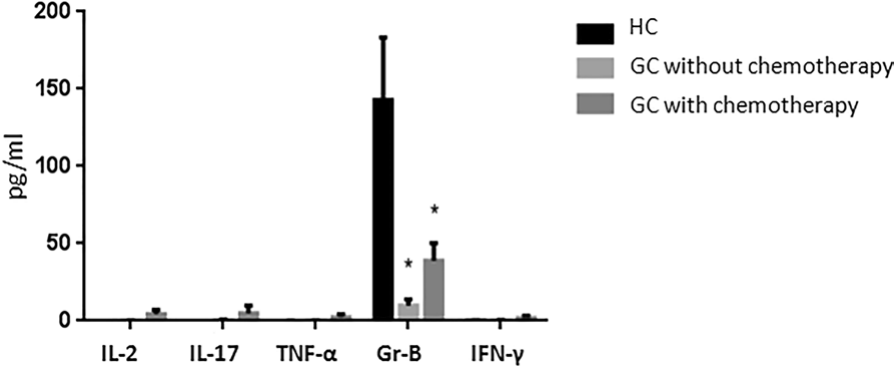
Detection and difference of MAIT cell-associated cytokines in serum from GC patients and HC. p < 0.05 were consider as statistically significant (*p < 0.05, **p < 0.01, ***p < 0.001)

On antigen recognition, MAIT cells can rapidly produce Th1/Th17 cytokines, including interferon (IFN)-γ, tumor necrosis factor (TNF)-α, interleukin (IL)-2 and interleukin (IL)-17, in an innate-like manner [16]. Samples were selected from 60 GC patients (30 cases were treated with chemotherapy, 30 were without) and 30 HC with age- and gender-matched. The levels of TNF-α, IFN-γ, IL-2, IL-17A and GrB in serum were determined by cytometric bead array (CBA). We demonstrated the cytokine levels of IL-2,IL-17A, IFN-γ and TNF-α were little or nothing detected in HC serum. Interestingly, high levels of serum GrB were detected in all groups. In comparison with HC, significantly lower level of GrB was founded in GC patients (mean 142.87/9.68, Fig. 5) serum. Together, these data also suggested that the level of GrB in GC patients with chemotherapy was lower than that without chemotherapy (mean 37.93/9.68, Fig. 5).

## Discussion

At present, cancer immunotherapy has an increasingly extensive application prospect. Peterfalvi et al. proposed for the first time that MAIT cells might be involved in the pathogenesis of malignant tumors. They tracked MAIT cells by SSCP and then discovered that MAIT cells could enter the kidney and brain [15]. Further studies found MAIT cells might be involved in the pathogenesis of tumor by secreting pro-inflammatory cytokines to change the local tumor microenvironment. Other literatures still reported that MAIT cells had obviously infiltrated into tumor tissues of kidney, brain and colon, suggesting MAIT cells might play a potential role in tumor immunity, and the gradual decline of peripheral blood MAIT cells might reflect the degree of cancer progression [15]. Still, studies had shown that the frequency of MAIT cells in patients with CRC is significantly decreased in peripheral blood, but increased in tumor tissues. And MAIT cells’ frequency in the tumor tissues was positively correlated with the serum CEA level in tumor patients. These datum indicated that activated and memory MAIT cells accumulated in CRC tumor tissues, regulating the internal development and progression of CRC. We speculated the same regulatory mechanism might also exist in GC, and subsequent studies in GC tissues may provide new insights into the role of MAIT cells in the regulatory process of GC. Our work had extensively described the characteristics of MAIT cells in GC patients. We focused on the frequency, partial function and clinical relevance of this cell in this study.

Firstly, in light of the MAIT cells significance in mucosal immunity [22], our experiments also analyzed the distribution of MAIT cells just in healthy population, which can be useful for studying on the MAIT cell level and partial function changes in other malignant diseases for monitoring some changes during these diseases. Our result showed the frequency of MAIT cells was negatively correlated with the increasing age and it had no difference in terms of gender in healthy individuals. Therefore, this conclusion supports the notion that it is necessary to pay attention to the age ratio in future studies with MAIT cell researches in diseases. Coupled with the incidence ratio of GC is 2:1 from male to female, our research also matched the gender ratio at the same time.

Furthermore, the frequency of peripheral blood MAIT cells were significantly lower than in HC. But no difference was found between GC patients with or without chemotherapy. It may be the reason that the blood samples from GC patients with chemotherapy were collected only 1–2 days after chemotherapy in the hospital, chemotherapy drugs had not yet changed the frequency of peripheral blood MAIT cells. Other reports also have pointed out the frequency of peripheral blood MAIT cells in tumor patients was lower than HC [12, 23]. As we all know, there were various phenotypes on the surface of MAIT cells, in addition to CD4, CD8, IL-12 receptors and IL-18 receptors, also includes chemokine receptors CCR5, CCR6 and CXCR6 [19, 24], and immune-related molecules NKG2D, PD-1 and CTLA-4 [19], etc. Some molecules are important in tumor immunity. For example, PD-1 is valuable for the study of tumor with MAIT cells, and we also determined the expression of PD-1 in peripheral blood MAIT cells, but had no significant positive result. The reason may be the number of cells was not enough, or the combination of antibodies was inappropriate. Checkpoint blockade therapy using anti-PD-1 and anti-CTLA-4 based drugs to inhibit tumor-mediated immunosuppression is proving to be a very powerful approach to treating some types of cancer [25]. Hence, it is suggested that future researchers who will explore tumors with immune cells should have a try to add the detection of PD-1 and CTLA-4. Moreover, NKG2D that expressed by MAIT cells is also well-known to play a key role in facilitating tumor immune surveillance [26]. The potential benefits of MAIT cell-based tumor immunotherapy need to be discussed.

Previous studies have shown IFN-γ is crucial for T cell immunity and can regulate the p53 signaling to induce tumor cell cycle arrest and apoptosis [27–29]. To go into the function of MAIT cells, we selected IFN-γ, TNF-α, IL-2 and IL-17A cytokines. Some literatures reported the peripheral blood MAIT cells produced almost exclusively IFN-γ and TNF-α, but IL-17A was almost undetectable in human peripheral blood [30]. Only with the stimulation, such as *E. coil*, significant IL-17A can be detected in peripheral blood [31]. In our experiments, Th1 cytokines (IFN-γ and TNF-α) were detected with the stimulation of PMA, ionomycin and monensin. However, Th17 cytokines were virtually undetected (IL-2 and IL-17A). And there was no difference in Th1 cytokines of MAIT cells between GC and HC.

Finally, MAIT cells are important immune cells, and playing an important role with staving off cancer in the immune response. Hence, we wanted to explore the correlation between the distribution of MAIT cells and clinical tumor parameters, to further explore the academic values of MAIT cells in GC. We collected the clinical parameters of serum CEA, CA19-9 and CA72-4 levels from 37 GC patients and performed Pearson correlation analysis with their frequency of MAIT cells in peripheral blood. We discovered no correlation. In addition, the GC patients serums were harvested and the levels of MAIT-associated cytokines were analyzed by CBA with the Human Th1/Th17 Cytometric Bead Array. We found Th1 and Th17 cytokines were rarely detected in HC. Only GrB, a molecule associated with MAIT cells, had the difference in GC patients and HC. In the next work, we will be interested in following up these patients and collecting their DFS and OS data for further to investigate the prognostic value of MAIT cells in this population.

Given that MAIT cells had been already proved to accumulate in inflamed mucosa, kidney and brain tumor tissues [27, 32, 33]. From our views, future studies can focus on exploring the distribution and function of MAIT cells in GC tissues and co-culture of MAIT cells with GC strains. Researches also had shown MAIT cells can kill target cells, including tumor cells, by cytotoxic particle threshing [34]. And MAIT cells may participate in the surveillance for CRC [12]. But until now, the role of MAIT cells in the development and progression of cancer is still unclear, which needs to be studied for further. Meanwhile, next investigations can put emphasis on addressing the clinical outcome(s) of novel MAIT cell-based interventions. In years to come, it can be studied from the aspects of tumor microenvironment and cell killing.

## Conclusions

Our study shows the decreased frequency, changed phenotypes and partial potentially impaired function of MAIT cells in GC patients. Considering the changes of MAIT cells in tumor patients peripheral blood, it will be suggested a possible MAIT cell-based immunological surveillance of GC. Moreover, it can provide relevant theoretical support for tumor immunotherapy.

## Data Availability

The datasets used and/or analysed during the current study are available from the corresponding author on reasonable request.

## Abbreviations

CA19-9: Carbohydrate antigen 19-9
CA72-4: Carbohydrate antigen 72-4
CBA: Cytometric bead array
CCR: C-C chemokine receptor
CEA: Carcinoembryonic antigen
CTLA-4: Cytotoxic Lymphocyte Antigen 4
CXCR: C-X-C chemokine receptor
DFS: Disease-free survival
GC: Gastric cancer
GrB: Granzyme-B
HC: Healthy controls
MAIT: Mucosal-associated invariant T cells
MR1: MHC-related 1
NKG2D: Natural-killer group 2, member D
OS: Overall survival
PBMC: Peripheral blood mononuclear cell
PD-1: Programmed cell death protein 1
TCR: T cell receptor
TH1: T helper 1
Th17: T helper 17
